# Supplementary material for: End Sequence Analysis Toolkit (ESAT) expands the extractable information from single-cell RNA-seq data
Source: Genome Res. 2016 Oct;26(10):1397–410. doi: 10.1101/gr.207902.116 (PMC5052061; doi:10.1101/gr.207902.116)
Supplement: Supplemental Material [file supp_gr.207902.116_Supp_Materials.tar.gz › ESAT-esat_v0.1/doc/html/classbroad_1_1core_1_1multiplealignment_1_1_multiple_alignment.html]

ESAT: broad.core.multiplealignment.MultipleAlignment Class Reference


ESAT


- Main Page
- Related Pages
- Packages
- Classes
- Files

- Class List
- Class Index
- Class Hierarchy
- Class Members

All Classes Namespaces Files Functions Variables Enumerator Pages

- broad
- core
- multiplealignment
- MultipleAlignment

Classes |
Public Member Functions |
Static Public Attributes |
Protected Member Functions |
Static Protected Attributes |
List of all members

broad.core.multiplealignment.MultipleAlignment Class Reference

Inheritance diagram for broad.core.multiplealignment.MultipleAlignment:

[legend]

|  |  |
| --- | --- |
| Classes | |
| class | **AlignedSequence** |
|  | |

|  |  |
| --- | --- |
| Public Member Functions | |
|  | MultipleAlignment () |
|  | |
|  | MultipleAlignment (List<?extends MultipleAlignment > alnBlocks, String ref, String[] seqs) |
|  | |
| void | encode () |
|  | |
| void | encode (boolean distinguishMissingSequence) |
|  | |
| void | encodeAsMatrix () |
|  | |
| void | reverse () |
|  | |
| int | length () |
|  | |
| Map< String, Short > | getColumn (int i) |
|  | |
| Map< String, Matrix > | getColumnsAsVector (int start, int number) throws ArrayIndexOutOfBoundsException |
|  | |
| Map< String, Matrix > | getAsMatrix () |
|  | |
| void | addShortEncodedColumn (Map< String, Short > col) |
|  | |
| void | addShortEncodedColumnMatrix (Map< String, Matrix > col) |
|  | |
| void | addShortEncodedRegion (Map< String, short[]> region) |
|  | |
| char | decodeShort (Short nucleotide) |
|  | |
| void | addSequence (AlignedSequence seq) |
|  | |
| void | addSequences (List< AlignedSequence > sequences) |
|  | |
| boolean | isEmpty () |
|  | |
| void | setIOHelper (MultipleAlignmentIO maio) |
|  | |
| void | write (BufferedWriter bw) throws IOException |
|  | |
| void | write (BufferedWriter bw, List< String > order) throws IOException |
|  | |
| MultipleAlignment | sampleColumns (int colNum, int numConsecutiveCols) |
|  | |
| void | permuteColumns () |
|  | |
| float | getScore () |
|  | |
| void | setScore (float score) |
|  | |
| AlignedSequence | getAlignedSequence (String containingSequenceId) |
|  | |
| List< AlignedSequence > | getAlignedSequences () |
|  | |
| List< String > | getAlignedSequenceIds () |
|  | |
| boolean | overlaps (SequenceRegion region) |
|  | |
| String | getReferenceId () |
|  | |
| void | setReferenceId (String referenceId) |
|  | |
| int | getReferenceStart () |
|  | |
| AlignedSequence | getReference () |
|  | |
| int | getReferenceEnd () |
|  | |
| boolean | isRefGapped () |
|  | |
| void | setRefGapped (boolean isRefGapped) |
|  | |
| MultipleAlignment | getConcatenatedSubAlignment (String refId, List<?extends GenomicAnnotation > annotations) |
|  | |
| void | compress () |
|  | |
| void | remove (List< String > removeList) |
|  | |
| MultipleAlignment | getSubAlignment (int refStart, int refEnd, boolean reverse) |
|  | |
| int | getNumberOfAligningSequences () |
|  | |
| MultipleAlignment | getSubAlignment (String refId, int refStart, int refEnd, boolean reverse) |
|  | |
| void | append (MultipleAlignment other) |
|  | |
| List< int[]> | getUngappedReferenceIslands () |
|  | |
| List< int[]> | getUngappedSequenceReferenceIslands () |
|  | |
| void | introduceGaps (boolean canGapReference, int maxNumberOfGaps) throws IllegalAccessException |
|  | |

|  |  |
| --- | --- |
| Static Public Attributes | |
| static final short | A\_CODE = Sequence.SHORT\_ENCODED\_A |
|  | |
| static final short | C\_CODE = Sequence.SHORT\_ENCODED\_C |
|  | |
| static final short | G\_CODE = Sequence.SHORT\_ENCODED\_G |
|  | |
| static final short | T\_CODE = Sequence.SHORT\_ENCODED\_T |
|  | |
| static final short | GAP\_CODE = Sequence.SHORT\_ENCODED\_GAP |
|  | |

|  |  |
| --- | --- |
| Protected Member Functions | |
| MultipleAlignmentIO | getIOHelper () |
|  | |
| Iterator< AlignedSequence > | getAlignedSequences (String refId, int refStart, int refEnd, boolean reverse) |
|  | |

|  |  |
| --- | --- |
| Static Protected Attributes | |
| static int | UNGAPPED\_ALPHABET\_SIZE = 4 |
|  | |

## Constructor & Destructor Documentation

|  |  |  |  |  |
| --- | --- | --- | --- | --- |
| broad.core.multiplealignment.MultipleAlignment.MultipleAlignment | ( |  | ) |  |

Here is the caller graph for this function:

|  |  |  |  |
| --- | --- | --- | --- |
| broad.core.multiplealignment.MultipleAlignment.MultipleAlignment | ( | List<?extends MultipleAlignment > | *alnBlocks*, |
|  |  | String | *ref*, |
|  |  | String[] | *seqs* |
|  | ) |  |  |

Constructs a multiple alignment object out of piecemeal multiple alignment blocks with, possible, different number of aligned sequences.

Parameters
:   |  |  |
    | --- | --- |
    | exonAlnBlocks | The blocks to combined into a single multiple alignment block |
    | string | reference sequence id |
    | seqs | array of sequence ids, the end alignment will include all even in the cases where a sequence alignment is gap only. |

## Member Function Documentation

|  |  |  |  |  |  |
| --- | --- | --- | --- | --- | --- |
| void broad.core.multiplealignment.MultipleAlignment.addSequence | ( | AlignedSequence | *seq* | ) |  |

Here is the caller graph for this function:

|  |  |  |  |  |  |
| --- | --- | --- | --- | --- | --- |
| void broad.core.multiplealignment.MultipleAlignment.addSequences | ( | List< AlignedSequence > | *sequences* | ) |  |

Here is the call graph for this function:

|  |  |  |  |  |  |
| --- | --- | --- | --- | --- | --- |
| void broad.core.multiplealignment.MultipleAlignment.addShortEncodedColumn | ( | Map< String, Short > | *col* | ) |  |

Here is the call graph for this function:

|  |  |  |  |  |  |
| --- | --- | --- | --- | --- | --- |
| void broad.core.multiplealignment.MultipleAlignment.addShortEncodedColumnMatrix | ( | Map< String, Matrix > | *col* | ) |  |

Here is the call graph for this function:

|  |  |  |  |  |  |
| --- | --- | --- | --- | --- | --- |
| void broad.core.multiplealignment.MultipleAlignment.addShortEncodedRegion | ( | Map< String, short[]> | *region* | ) |  |

Here is the call graph for this function:

|  |  |  |  |  |  |
| --- | --- | --- | --- | --- | --- |
| void broad.core.multiplealignment.MultipleAlignment.append | ( | MultipleAlignment | *other* | ) |  |

Here is the call graph for this function:

|  |  |  |  |  |
| --- | --- | --- | --- | --- |
| void broad.core.multiplealignment.MultipleAlignment.compress | ( |  | ) |  |

Compresses alignment by removing columns containing reference gaps

Here is the call graph for this function:

|  |  |  |  |  |  |
| --- | --- | --- | --- | --- | --- |
| char broad.core.multiplealignment.MultipleAlignment.decodeShort | ( | Short | *nucleotide* | ) |  |

Here is the caller graph for this function:

|  |  |  |  |  |
| --- | --- | --- | --- | --- |
| void broad.core.multiplealignment.MultipleAlignment.encode | ( |  | ) |  |

|  |  |  |  |  |  |
| --- | --- | --- | --- | --- | --- |
| void broad.core.multiplealignment.MultipleAlignment.encode | ( | boolean | *distinguishMissingSequence* | ) |  |

|  |  |  |  |  |
| --- | --- | --- | --- | --- |
| void broad.core.multiplealignment.MultipleAlignment.encodeAsMatrix | ( |  | ) |  |

|  |  |  |  |  |  |
| --- | --- | --- | --- | --- | --- |
| AlignedSequence broad.core.multiplealignment.MultipleAlignment.getAlignedSequence | ( | String | *containingSequenceId* | ) |  |

Here is the caller graph for this function:

|  |  |  |  |  |
| --- | --- | --- | --- | --- |
| List<String> broad.core.multiplealignment.MultipleAlignment.getAlignedSequenceIds | ( |  | ) |  |

Here is the caller graph for this function:

|  |  |  |  |  |
| --- | --- | --- | --- | --- |
| List<AlignedSequence> broad.core.multiplealignment.MultipleAlignment.getAlignedSequences | ( |  | ) |  |

Here is the caller graph for this function:

|  |  |  |  |
| --- | --- | --- | --- |
| Iterator<AlignedSequence> broad.core.multiplealignment.MultipleAlignment.getAlignedSequences | ( | String | *refId*, |
|  |  | int | *refStart*, |
|  |  | int | *refEnd*, |
|  |  | boolean | *reverse* |
|  | ) |  |  |

protected

|  |  |  |  |  |
| --- | --- | --- | --- | --- |
| Map<String, Matrix> broad.core.multiplealignment.MultipleAlignment.getAsMatrix | ( |  | ) |  |

Here is the call graph for this function:

|  |  |  |  |  |  |
| --- | --- | --- | --- | --- | --- |
| Map<String, Short> broad.core.multiplealignment.MultipleAlignment.getColumn | ( | int | *i* | ) |  |

Assumes that encode has been called and each aligned sequence is encoded

Parameters
:   |  |  |
    | --- | --- |
    | int | - alignment column |

Here is the call graph for this function:

|  |  |  |  |
| --- | --- | --- | --- |
| Map<String, Matrix> broad.core.multiplealignment.MultipleAlignment.getColumnsAsVector | ( | int | *start*, |
|  |  | int | *number* |
|  | ) |  | throws ArrayIndexOutOfBoundsException |

Here is the call graph for this function:

|  |  |  |  |
| --- | --- | --- | --- |
| MultipleAlignment broad.core.multiplealignment.MultipleAlignment.getConcatenatedSubAlignment | ( | String | *refId*, |
|  |  | List<?extends GenomicAnnotation > | *annotations* |
|  | ) |  |  |

Here is the call graph for this function:

|  |  |  |  |  |
| --- | --- | --- | --- | --- |
| MultipleAlignmentIO broad.core.multiplealignment.MultipleAlignment.getIOHelper | ( |  | ) |  |

protected

Here is the caller graph for this function:

|  |  |  |  |  |
| --- | --- | --- | --- | --- |
| int broad.core.multiplealignment.MultipleAlignment.getNumberOfAligningSequences | ( |  | ) |  |

Computes the number of sequences that have aligning bases as opposed to being pure gap

Returns
:   Number of sequences that are not gap only.

Here is the call graph for this function:

|  |  |  |  |  |
| --- | --- | --- | --- | --- |
| AlignedSequence broad.core.multiplealignment.MultipleAlignment.getReference | ( |  | ) |  |

Here is the call graph for this function:

Here is the caller graph for this function:

|  |  |  |  |  |
| --- | --- | --- | --- | --- |
| int broad.core.multiplealignment.MultipleAlignment.getReferenceEnd | ( |  | ) |  |

Here is the call graph for this function:

|  |  |  |  |  |
| --- | --- | --- | --- | --- |
| String broad.core.multiplealignment.MultipleAlignment.getReferenceId | ( |  | ) |  |

Here is the caller graph for this function:

|  |  |  |  |  |
| --- | --- | --- | --- | --- |
| int broad.core.multiplealignment.MultipleAlignment.getReferenceStart | ( |  | ) |  |

Here is the call graph for this function:

Here is the caller graph for this function:

|  |  |  |  |  |
| --- | --- | --- | --- | --- |
| float broad.core.multiplealignment.MultipleAlignment.getScore | ( |  | ) |  |

|  |  |  |  |
| --- | --- | --- | --- |
| MultipleAlignment broad.core.multiplealignment.MultipleAlignment.getSubAlignment | ( | int | *refStart*, |
|  |  | int | *refEnd*, |
|  |  | boolean | *reverse* |
|  | ) |  |  |

Here is the call graph for this function:

Here is the caller graph for this function:

|  |  |  |  |
| --- | --- | --- | --- |
| MultipleAlignment broad.core.multiplealignment.MultipleAlignment.getSubAlignment | ( | String | *refId*, |
|  |  | int | *refStart*, |
|  |  | int | *refEnd*, |
|  |  | boolean | *reverse* |
|  | ) |  |  |

Parameters
:   |  |  |
    | --- | --- |
    | refId | - The sequence id in which coordinates are given |
    | refStart | - Start of region of interest in the refId sequence. Positions start in 1. |
    | refEnd | - End of region of interest in the refId sequence. Positions start in 1. |
    | reverse | - If true the multiple alignment will return all sequences in the opposite strand |

Returns
:   The multiple alignment of the region of interest or an empty alignment if the coordinates do not overlap the refId aligned region.

Here is the call graph for this function:

|  |  |  |  |  |
| --- | --- | --- | --- | --- |
| List<int[]> broad.core.multiplealignment.MultipleAlignment.getUngappedReferenceIslands | ( |  | ) |  |

Finds the list of ungapped regions in the reference sequence.

Returns
:   A list of two sized integer arrays with the start and end of the ungapped regions. The convention here is semi closed intervals, each list items is of the form [start, end).

Here is the call graph for this function:

Here is the caller graph for this function:

|  |  |  |  |  |
| --- | --- | --- | --- | --- |
| List<int[]> broad.core.multiplealignment.MultipleAlignment.getUngappedSequenceReferenceIslands | ( |  | ) |  |

Finds the list of ungapped sequence (as opposed to alignment gaps) regions in the reference sequence.

Returns
:   A list of two sized integer arrays with the start and end of the ungapped regions. The convention here is semi closed intervals, each list items is of the form [start, end).

Here is the call graph for this function:

|  |  |  |  |
| --- | --- | --- | --- |
| void broad.core.multiplealignment.MultipleAlignment.introduceGaps | ( | boolean | *canGapReference*, |
|  |  | int | *maxNumberOfGaps* |
|  | ) |  | throws IllegalAccessException |

Here is the call graph for this function:

|  |  |  |  |  |
| --- | --- | --- | --- | --- |
| boolean broad.core.multiplealignment.MultipleAlignment.isEmpty | ( |  | ) |  |

Here is the caller graph for this function:

|  |  |  |  |  |
| --- | --- | --- | --- | --- |
| boolean broad.core.multiplealignment.MultipleAlignment.isRefGapped | ( |  | ) |  |

Here is the caller graph for this function:

|  |  |  |  |  |
| --- | --- | --- | --- | --- |
| int broad.core.multiplealignment.MultipleAlignment.length | ( |  | ) |  |

Here is the caller graph for this function:

|  |  |  |  |  |  |
| --- | --- | --- | --- | --- | --- |
| boolean broad.core.multiplealignment.MultipleAlignment.overlaps | ( | SequenceRegion | *region* | ) |  |

|  |  |  |  |  |
| --- | --- | --- | --- | --- |
| void broad.core.multiplealignment.MultipleAlignment.permuteColumns | ( |  | ) |  |

Here is the call graph for this function:

|  |  |  |  |  |  |
| --- | --- | --- | --- | --- | --- |
| void broad.core.multiplealignment.MultipleAlignment.remove | ( | List< String > | *removeList* | ) |  |

Here is the call graph for this function:

|  |  |  |  |  |
| --- | --- | --- | --- | --- |
| void broad.core.multiplealignment.MultipleAlignment.reverse | ( |  | ) |  |

|  |  |  |  |
| --- | --- | --- | --- |
| MultipleAlignment broad.core.multiplealignment.MultipleAlignment.sampleColumns | ( | int | *colNum*, |
|  |  | int | *numConsecutiveCols* |
|  | ) |  |  |

Here is the call graph for this function:

|  |  |  |  |  |  |
| --- | --- | --- | --- | --- | --- |
| void broad.core.multiplealignment.MultipleAlignment.setIOHelper | ( | MultipleAlignmentIO | *maio* | ) |  |

|  |  |  |  |  |  |
| --- | --- | --- | --- | --- | --- |
| void broad.core.multiplealignment.MultipleAlignment.setReferenceId | ( | String | *referenceId* | ) |  |

Here is the caller graph for this function:

|  |  |  |  |  |  |
| --- | --- | --- | --- | --- | --- |
| void broad.core.multiplealignment.MultipleAlignment.setRefGapped | ( | boolean | *isRefGapped* | ) |  |

Here is the call graph for this function:

|  |  |  |  |  |  |
| --- | --- | --- | --- | --- | --- |
| void broad.core.multiplealignment.MultipleAlignment.setScore | ( | float | *score* | ) |  |

|  |  |  |  |  |  |
| --- | --- | --- | --- | --- | --- |
| void broad.core.multiplealignment.MultipleAlignment.write | ( | BufferedWriter | *bw* | ) | throws IOException |

|  |  |  |  |
| --- | --- | --- | --- |
| void broad.core.multiplealignment.MultipleAlignment.write | ( | BufferedWriter | *bw*, |
|  |  | List< String > | *order* |
|  | ) |  | throws IOException |

## Member Data Documentation

final short broad.core.multiplealignment.MultipleAlignment.A\_CODE = Sequence.SHORT\_ENCODED\_A

static

final short broad.core.multiplealignment.MultipleAlignment.C\_CODE = Sequence.SHORT\_ENCODED\_C

static

final short broad.core.multiplealignment.MultipleAlignment.G\_CODE = Sequence.SHORT\_ENCODED\_G

static

final short broad.core.multiplealignment.MultipleAlignment.GAP\_CODE = Sequence.SHORT\_ENCODED\_GAP

static

final short broad.core.multiplealignment.MultipleAlignment.T\_CODE = Sequence.SHORT\_ENCODED\_T

static

int broad.core.multiplealignment.MultipleAlignment.UNGAPPED\_ALPHABET\_SIZE = 4

staticprotected

---

The documentation for this class was generated from the following file:

- java/broad/core/multiplealignment/MultipleAlignment.java


---

Generated on Fri Aug 15 2014 15:10:55 for ESAT by  

 1.8.7
